# Relationship between work-family conflict and anxiety/depression among Chinese correctional officers: a moderated mediation model of burnout and resilience

**DOI:** 10.1186/s12889-023-17514-6

**Published:** 2024-01-02

**Authors:** Ying Huang, Huijuan Guo, Siyuan Wang, Shaoling Zhong, Yuqiong He, Hui Chen, Jiansong Zhou, Xiaoping Wang

**Affiliations:** 1 Department of Psychiatry, National Clinical Research Center for Mental Disorders, and National Center for Mental Disorders, Hunan Key Laboratory of Psychiatry and Mental Health, The Second Xiangya Hospital of Central South University, National Technology Institute on Mental Disorders, No. 139 Middle Renmin Road, Changsha, Hunan Province 410011 China; 2 Pingtang Compulsory Isolation Detoxification Institute in Hunan Province, Changsha, China; 3 Department of Community Mental Health, the Affiliated Brain Hospital of Guangzhou Medical University, Guangzhou, China

**Keywords:** Correctional officer, Work-family conflict, Psychological distress, Burnout, Resilience, Occupational health

## Abstract

**Background:**

Correctional officers tend to have high levels of work-family conflict (WFC). WFC has been found associated with various forms of psychological distress and to affect the overall well-being of correctional officers. Burnout and resilience may affect the relationship between WFC and psychological distress, however, this association still remains unclear. This study aimed to examine the mediating effect of burnout on the relationship between WFC and anxiety/depression and the moderating role of resilience, within the context of correctional officers.

**Methods:**

A cross-sectional online survey was conducted in China from October 2021 to January 2022. WFC, burnout, resilience, anxiety, and depression were evaluated using the Work-Family Conflict Scale (WFCS), Maslach Burnout Inventory-General Survey (MBI-GS), 10-item Connor-Davidson Resilience Scale (CD-RISC-10), and the Depression Anxiety Stress Scale (DASS). Mediation and moderation models were then tested using the PROCESS macro in SPSS, with burnout being a mediator and resilience playing a moderating role in the relationship between WFC and anxiety/depression.

**Results:**

A total of 472 correctional officers were included. Burnout was found to mediate the relationship between WFC and anxiety (*b* = 0.14, 95%CI [0.10, 0.19]) and the relationship between WFC and depression (*b* = 0.23, 95%CI [0.18, 0.28]). Additionally, resilience played a moderating role in the direct effect of WFC on anxiety (*b* = − 0.02, *p* < 0.01) and the first half of the indirect effect of WFC on anxiety (*b* = − 0.007, *p* < 0.05). Furthermore, resilience was also found to moderate the first half of the indirect effect of WFC on depression (*b* = − 0.02, p < 0.01), but not the direct effect of WFC on depression (*b* = − 0.005, *p* > 0.05).

**Conclusion:**

The findings of the present study may improve our understanding by elucidating the fundamental mechanisms of the connection between WFC and psychological distress among correctional officers. The results have significant implications for policymakers and individuals, as they suggest that diverse interventions may help promote the mental well-being of correctional officers.

**Supplementary Information:**

The online version contains supplementary material available at 10.1186/s12889-023-17514-6.

## Introduction

### Background

The job of a correctional officer has been recognized as highly demanding and burdensome [1]. Correctional officers are exposed to great occupational stress, misunderstanding, danger and unexcepted emergencies [2, 3], which makes them more susceptible to somatic problems and psychological distress compared to the general population and people with other occupations [4, 5]. Generally, the most common psychological presentations of correctional officers are anxiety and depression [6, 7]. A comprehensive review of surveys conducted among correctional officers in six countries revealed that the prevalence of anxiety and depression could be as high as 12.2–37.9% and 24%-59.7%, respectively [7]. A recent investigation in China during the COVID-19 pandemic indicated that the prevalence of anxiety and depression among correctional officers reached 72.6% and 69.8%, respectively [6]. The presence of anxiety and depression not only impacts the work performance of correctional officers but is also associated with their increased suffering and suicidal risk [5]. Therefore, it is imperative to investigate the risk factors associated with psychological distress among correctional officers and the underlying mechanisms that contribute to its development.

Work-family conflict (WFC), a prevalent stressor for correctional officers [8], has been found associated with various forms of psychological distress, including anxiety, depression, psychological strain, and somatic symptoms [8, 9]. Due to the unique nature of correctional work and the work environment, correctional officers are often faced with challenging tasks and schedules that are constantly changing, which require a significant amount of time and effort [10]. Correctional officers may also have family responsibilities; thus, the challenge of balancing their work and personal life makes them more susceptible to a higher level of WFC [11]. Although WFC has become a prominent problem affecting the mental well-being of correctional officers [8, 12], relatively few studies have examined the association between WFC and psychological distress in this population as well as the underlying mechanism that mediate or moderate this relationship. The present study aims to explore the mediator, burnout, and the moderator, resilience, between WFC and anxiety/depression among Chinese correctional officers.

### Literature review and development of hypotheses

#### WFC and psychological distress

WFC can be characterized as an inter-role conflict arising from incongruity between the demands of work and family roles [13]. The role strain theory indicates that the combination of multiple roles, such as an employee and a family caregiver, can increase work strain and lead to negative health outcomes [14]. Numerous studies on different occupational populations have suggested that a high level of WFC is associated with high levels of mood disturbance, anxiety, burnout and other psychological distresses [15, 16]. This association was also found in correctional officers [9, 17]. For example, a study found that WFC was significantly and positively associated with depression among correctional officers [9]. A recent Chinese study showed that correctional officers experiencing significant WFC exhibited high levels of anxiety and depressive symptoms [6]. However, the literature on the relationship between WFC and psychological distress among correctional officers is still limited, necessitating further studies on this relationship and the mechanism.

#### Burnout as a mediator

Burnout is a prolonged response to chronic emotional and interpersonal stressors at work, and it includes three dimensions, namely emotional exhaustion, depersonalization and personal accomplishment [18]. The Job Demands-Resources model (JD-R model) posits that WFC, as a chronic stressor, can deplete the physical, psychological and emotional resources of individuals, ultimately resulting in burnout [19]. Consistent with this theoretical framework, prior studies focusing on police officers have indicated a positive correlation between WFC and burnout [20]. Researchers have also identified WFC as a significant predictor of a higher level of stress and burnout among police officers [8].

Moreover, empirical evidence has demonstrated that workplace burnout can lead to adverse emotional states, which may affect the mental well-being of individuals if it is prolonged [21]. Numerous studies conducted on diverse occupational cohorts have consistently indicated the negative effect of burnout on mental health [21, 22]. For instance, a longitudinal study revealed that early burnout significantly predicted depressive symptoms in later stages of life [23]. Furthermore, researchers have identified a positive association between elevated levels of WFC and heightened anxiety levels, with emotional exhaustion serving as a crucial mediator in this relationship [24]. Based on the above studies, we speculated that burnout might be a mediator of the relationship between WFC and psychological distress among correctional officers in China.

#### Resilience as a moderator

Resilience is defined as the ability to cope and adapt when faced with major stressors such as life adversity, traumas, tragedies and threats [25]. As an important psychological resource, resilience helps to alleviate the impact of negative events and their adverse consequences, thus playing a protective role in the mental health of individuals [26]. Numerous prior studies have identified the moderating effect of resilience on WFC across various occupational cohorts [27, 28]. For instance, a study revealed that resilience played a significant role in moderating the adverse impact of WFC on the mental health of healthcare workers [29]. Another study indicated that teachers with a higher level of resilience could better manage their stress, allocate their resources, and manage their work-family dynamics, thereby alleviating burnout [30]. There are also studies showing the role of resilience in buffering against psychological distress, even in the presence of burnout [27]. Overall, the existing theoretical and empirical evidence suggests that individuals with high levels of resilience are more likely to maintain psychological stability and successfully adapt to their environment, thereby reducing the likelihood of developing negative mental health outcomes [31]. Thus, it is hypothesized that resilience may be a protective factor for psychological distress resulting from WFC, and that resilience may also moderate the link between WFC and burnout and the association between burnout and psychological distress.

#### Research gap and the present study

**Fig. 1:**
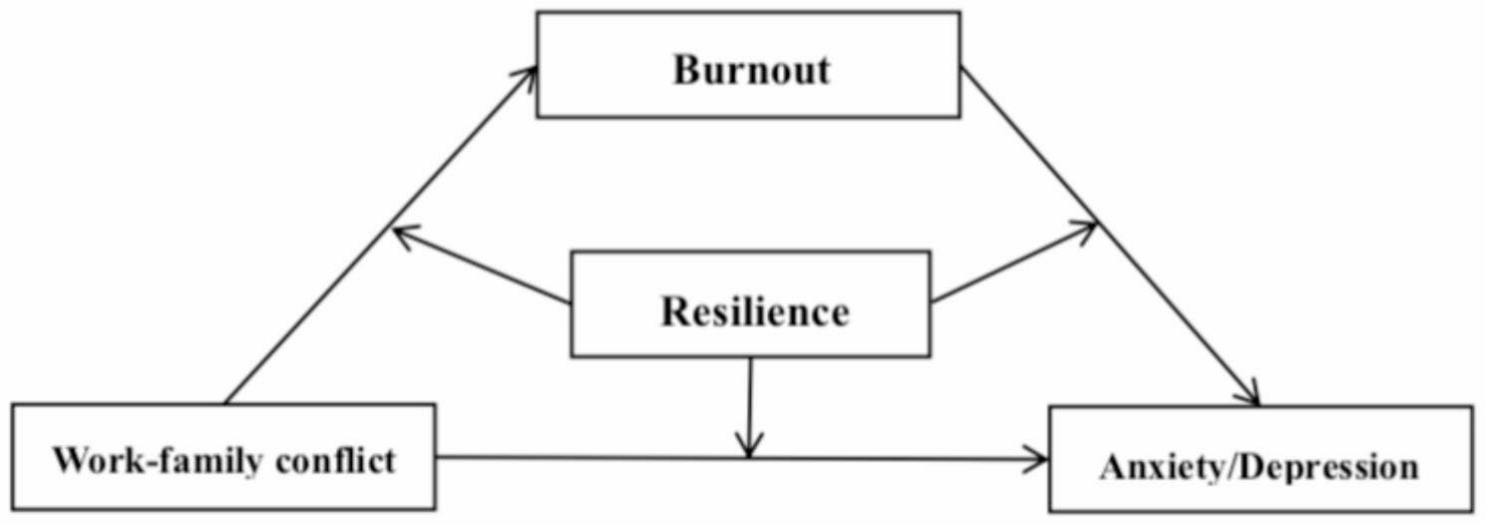
Conceptual model

Numerous studies have focused on the detrimental effects of WFC on the mental well-being of individuals. However, these studies have primarily centered on healthcare workers and teachers, with limited attention given to correctional officers, particularly in China. Therefore, it is crucial to address this research gap by focusing on WFC and the mental health of correctional officers. Additionally, the role of positive and negative psychological factors (e.g., resilience and burnout, respectively) in the relationship between WFC and psychological distress also remains largely unknown. To improve our understanding of these mechanisms, drawing upon the J-DR model and the evidence supporting the protective nature of resilience, we proposed a moderated mediating model with burnout as a mediating variable and resilience as a moderating variable (Fig. 1). Our objective was to examine whether burnout plays an intermediary role in the relationship between WFC and anxiety/depression and whether resilience moderates the intermediary process in which WFC affects the anxiety/depression. Therefore, we proposed the following hypotheses: (1) WFC may be positively associated with anxiety and depression; (2) burnout may play a mediation role in the association between WFC and anxiety/depression, i.e., increased WFC may be associated with a higher level of burnout, which leads to further increase in anxiety and depression; and (3) the direct effect of WFC on anxiety and depression may be moderated by resilience, and the indirect effect of burnout on the association between WFC and anxiety/depression may also be moderated by resilience.Exploring these problems is of great significance for the understanding of the mechanisms by which WFC impacts psychological distress.

## Methods

### Participants and procedure

This cross-sectional study was conducted between October 2021 and January 2022, with the target population being correctional officers from different provinces of China. An anonymous online questionnaire was prepared using Questionnaire Star (https://www.wjx.cn), a commonly used online survey platform in China. First, 10 correctional officers who attended an annual training program organized by the prison administration department for professional development and knowledge exchange were chosen as original deliverers. The criteria for the selection of original deliverers were as follows: (1) they volunteered to participate in this survey and were willing to recommend this survey to others, and (2) they were from a variety of provinces in China, ensuring the inclusion of correctional officers from diverse regions across China. Then, the link to the online questionnaire was shared on commonly used social media platforms, such as WeChat, and was distributed by the original deliverers to their friends and WeChat group members. The first section of the questionnaire included a concise overview of the study, along with assurances of anonymity and confidentiality. Before the start of the questionnaire, participants were required to provide informed consent by selecting the “agree” option; they were also given the option to discontinue the survey by selecting “disagree” and withdraw their participation at any time. This study was approved by the Ethics Committee of the Second Xiangya Hospital of Central South University.

### Sample size

The sample size was determined using an online calculator (http://www.raosoft.com/samplesize.html). Considering an estimated population of 400,000 correctional officers in China, a confidence level of 95%, an accuracy level of 5%, and a response distribution of 50%, the minimum sample size was determined to be 384.

### Measures

#### Sociodemographic characteristics

The sociodemographic information of all participants, including age, gender, level of education, years worked as a correctional officer, marital status and location of practice, were collected through forced-choice questions in the survey.

#### Work-family conflict

WFC experienced by participants was assessed using the Chinese version of the Work-Family Conflict Scale (WFCS) [32], which was developed by Carlson [33]. The WFCS consists of 18 items, which are divided into two subscales, i.e., work interference with family and family interference with work. All the items are rated on a 5-point Likert scale (from 1 = never to 5 = always), with the total score ranging from 18 to 90. For this scale, a higher total score indicates greater WFC. The internal consistency of the scale was 0.84, according to a study by Lu et al. using a sample of employees based in Taiwan [32]. The Cronbach’s α coefficient of this scale was 0.917 in this study.

#### Anxiety and depression

Symptoms of anxiety and depression were assessed using the Chinese version of the Depression Anxiety Stress Scale (DASS) [34]. DASS is a 21-item self-report questionnaire consisting of three subscales (depression, anxiety and stress), with seven items in each subscale; it assesses symptoms of depression, anxiety and stress over the past week. All items are rated on a 4-point Likert scale (from 0 = completely non-compliant to 3 = completely compliant). For each subscale, the total score is obtained by summing up the raw scores of corresponding items and multiplying the sum by 2, with higher scores indicating higher severity of symptoms. The internal consistency of the scale was 0.89, according to a study by Gong et al. on Chinese college students [34]. The Cronbach’s α coefficient for this scale was 0.952 in this study.

#### Burnout

A revised Chinese version of the Maslach Burnout Inventory-General Survey (MBI-GS) [35] was used to assess the burnout of participants. This inventory includes 15 items and assesses three dimensions of burnout: emotional exhaustion, depersonalization and personal accomplishment. All items are rated on a 7-point Likert scale (from 0 = never to 6 = every day), with five items for personal accomplishment being reverse-scored. The total score is calculated by summing up the scores of all items, which is divided by 15 and then multiplied by 20, resulting in a modified score of 0-120. For this scale, a higher score indicates greater burnout. The test-retest reliability (ICC = 0.71) of the scale was demonstrated acceptable in prior studies [36]. It has also been reported that the Cronbach’s α coefficient of the scales and dimensions ranged from 0.672 to 0.874 in Chinese nurses [36]. The Cronbach’s α coefficient for this scale was 0.830 in this study.

#### Resilience

The 10-item Connor-Davidson Resilience Scale (CD-RISC-10) [37], which was develop by Campbell-Sills and Stein, was used to measure the participants’ resilience. All items are rated on a *5*-point Likert scale (from 0 = never to 4 = always), with a total score ranging from 0 to 40; a higher total score represents greater resilience. The Chinese version also demonstrated favorable internal consistency (Cronbach’s alpha = 0.91) and test-retest reliability (r = 0.90) [38], and the Cronbach’s α coefficient for this scale was 0.945 in this study.

### Statistical analysis

Descriptive analysis was first performed on the sociodemographic characteristics and psychological variables of the participants. Categorical variables were presented as percentages, and continuous variables were presented as means (SDs). Pearson’s correlation analysis was performed to determine the association between WFC, resilience, burnout, anxiety and depression. The mediation role of burnout in the relationship between WFC and anxiety/depression was evaluated using the simple mediation model (model 4 of PROCESS macro) [39]. For both depression and anxiety, the mediating effect of burnout was determined using bootstrap 95% confidence intervals (5,000 samples), with a 95% CI that did not contain 0 indicating a significant mediating effect. Then, model 59 of PROCESS macro was used to test the moderating effect of resilience on the direct and indirect effects of WFC on anxiety/depression. Similarly, a 95% CI not including 0 was suggestive of a significant moderating effect on the mediation. A simple slopes analysis and the Johnson-Neyman technique were also used to examine the interactions between factors, with age, gender, and level of education included as covariates. All the analyses were performed using the SPSS software (Version 26.0; IBM), and the PROCESS macro for SPSS (version 3.4) was used to establish the mediation and moderated mediation models [39]. For all the analyses, two-tailed *p* < 0.05 indicated statistical significance.

## Results

### Sociodemographic characteristics

A total of 472 correctional officers employed in urban settings across 29 provincial administrative regions of China were included in this online survey. Participants from Tianjin, Tibet, Taiwan, Hong Kong, and Macao were excluded from the analysis due to their lack of response. All participants submitted completed answers, which ensured the reliability of the data. Among the participants, 62% (n = 294) were from Hunan Province, 4% (n = 17) were from Henan Province, 3% (n = 15) were from the Inner Mongolia Autonomous Region, and the remaining 31% (n = 146) were from the other 26 provinces. The geographic distribution of the participants is presented in ***Supplementary Material***. The mean age of the respondents was 37.63 ± 8.10 years, and their average work experience was 13.55 ± 9.48 years. Among all respondents, 31.8% were female and 68.2% were male; 15% were unmarried, 80.3% were married, and 4.7% were divorced; Regarding the level of education, 13.6% had completed junior college education and below, 80.5% completed their undergraduate education, and 5.9% had a postgraduate degree or above (Table 1).

**Table 1:** Sociodemographic information of participants (N = 472)

Variable		M ± SD / n (%)
Age (year)		37.63 ± 8.10
Work experience (year)		13.55 ± 9.48
Gender		
	Male	322 (68.2%)
	Female	150 (31.8%)
Marital status		
	Unmarried	71 (15.0%)
	Married	379 (80.3%)
	Divorced	22 (4.7% )
Level of education		
	Junior college and below	64 (13.6%)
	Undergraduate	380 (80.5%)
	Postgraduate and above	28 (5.9%)

M: mean, SD: standard deviation

### Preliminary analyses

The means, SDs, and Pearson correlations of the variables are presented in Table 2. The results indicated that WFC was positively associated with depression (*r* = 0.44, *p* < 0.01), anxiety (*r* = 0.43, *p* < 0.01) and burnout (*r* = 0.50, *p* < 0.01) but negatively correlated with resilience (*r* = − 0.29, *p* < 0.01). Burnout was positively correlated with depression (*r* = 0.68, *p* < 0.01) and anxiety (*r* = 0.55, *p* < 0.01) but negatively correlated with resilience (*r* = − 0.61, *p* < 0.01). Resilience was found negatively correlated with depression (*r* = − 0.52, *p* < 0.05) and anxiety (*r* = − 0.45, *p* < 0.01), and depression was positively correlated with anxiety (*r* = 0.68, *p* < 0.01). According to the effect size criteria proposed by Cohen (1992) [40] for correlation coefficients (ρ = 0.1 indicates a small effect size, ρ = 0.3 indicates a medium effect size, ρ = 0.5 indicates a large effect size), the minimum correlation coefficient was − 0.29 in this study, indicating close proximity to a medium effect size. Furthermore, the effect sizes of the remaining correlation coefficients exceeded the threshold for a moderate effect size.

**Table 2:** Correlations between WFC, burnout, resilience, depression and anxiety (N = 472)

	M	SD	WFC	Burnout	Resilience	Depression	Anxiety
WFC	60.45	12.90	1				
Burnout	51.47	27.00	0.50**	1			
Resilience	24.69	7.35	-0.29**	-0.61**	1		
Depression	13.69	9.60	0.44**	0.68**	-0.52*	1	
Anxiety	12.11	8.27	0.43**	0.55**	-0.45**	0.68**	1

WFC: work-family conflict, M: mean, SD: standard deviations, *p < 0.05 (two-tailed), **p < 0.01 (two-tailed)

### Mediation analyses

The results of mediation analyses showed that WFC was directly associated with anxiety (*b* = 0.12, *p* < 0.001) and indirectly related to anxiety through the mediation of burnout (*b* = 0.15, *p* < 0.001) (Table 3). The 95% bias-corrected bootstrap confidence interval also indicated significant indirect effects of burnout on the association between WFC and anxiety (*b* = 0.14, 95%CI [0.10, 0.19]). Taken together. the above results suggested that burnout might partially mediate the association between WFC and anxiety. Similar results were obtained through the mediation analyses with depression as the dependent variable, which showed that WFC was significantly and directly associated with depression (*b* = 0.09, *p* < 0.01) and that WFC was also significantly and indirectly related to depression through the mediation of burnout (*b* = 0.23, *p* < 0.001). The 95% bias-corrected bootstrap confidence interval for the indirect effect on the relationship between WFC and depression did not include 0 (*b* = 0.23, 95%CI [0.18, 0.28]), indicating that the indirect effect was significant.

**Table 3:** Mediation analyses

Predictor	Model 1 (outcome: burnout)				Model 2 (outcome: anxiety)				Model 3 (outcome: depression)			
	B	SE	t	95%CI	B	SE	t	95%CI	B	SE	t	95%CI
Age	-0.47	0.13	-3.56***	[-0.73, -0.21]	0.03	0.04	0.76	[-0.05, 0.11]	0.11	0.04	2.84**	[0.04, 0.19]
Gender	-1.94	2.35	-0.82	[-6.55, 2.68]	0.35	0.68	0.51	[-0.99, 1.69]	-0.48	0.70	-0.68	[-1.85, 0.89]
Level of education	4.49	2.51	1.79	[-0.45, 9.43]	-0.58	0.73	-0.79	[-2.02, 0.86]	-1.08	0.75	-1.44	[-2.55, 0.39]
WFC	0.98	0.08	11.68***	[0.82, 1.15]	0.12	0.03	4.37***	[0.07, 0.18]	0.09	0.03	3.31**	[0.04, 0.15]
Burnout					0.15	0.01	10.86***	[0.12, 0.17]	0.23	0.01	16.74***	[0.20, 0.26]
R^2^	0.27				0.34				0.49			
F	42.79***				48.55***				90.71***			

WFC: work-family conflict, M: mean, SD: standard deviation, **p < 0.01 (two-tailed), ***p < 0.001 (two-tailed)

### Moderated mediation analyses

The results of the moderated mediation analysis are presented in Table 4, In this analysis, WFC, anxiety, burnout and resilience were used as the predictor, dependent variable, mediator and moderator, respectively. The results showed that resilience had a moderating effect on the direct impact of WFC on anxiety (*b* = − 0.02, *p* < 0.01) and the first half of this impact mediated by the burnout (*b* = − 0.007, *p* < 0.05), but had no moderating effect on the second half of this impact mediated by burnout (*b* = − 0.003, *p* > 0.05). With regard to the impact of WFC on depression, when resilience was involved as a moderator, the moderated mediation analysis showed that resilience had a moderating effect on the first half of the impact mediated by burnout (*b* = − 0.02, p < 0.01), but had no moderating effect on the second half of the impact mediated by burnout (*b* = − 0.003, *p* > 0.05) or the direct impact of WFC on depression (*b* = − 0.005, *p* > 0.05).

**Table 4:** Moderated mediation analyses

Predictor	Model 1 (outcome: burnout)				Model 2 (outcome: anxiety)				Model 3 (outcome: depression)			
	B	SE	t	95%CI	B	SE	t	95%CI	B	SE	t	95%CI
Age	-0.38	0.11	-3.47**	[-0.60, -0.17]	0.008	0.04	0.20	[-0.07, 0.08]	0.09	0.04	2.36*	[0.02, 0.17]
Gender	-2.03	1.94	-1.04	[-5.84, 1.79]	0.31	0.66	0.47	[-0.99, 1.62]	-0.52	0.68	-0.76	[-1.86, 0.83]
Level of education	4.41	2.08	2.12*	[0.33, 8.49]	-0.50	0.71	-0.71	[-1.90, 0.90]	-1.01	0.73	-1.37	[-2.45, 0.44]
WFC	0.67	0.07	9.21***	[0.53, 0.81]	0.12	0.03	4.48***	[0.07, 0.17]	0.09	0.03	3.34**	[0.04, 0.15]
Resilience	-1.88	0.13	-14.79***	[-2.13, -1.63]	-0.24	0.05	-4.51***	[-0.34, -0.13]	-0.21	0.05	-3.86**	[-0.32, -0.10]
WFC×resilience	-0.02	0.008	-2.76**	[-0.04, -0.006]	-0.007	0.003	-2.31*	[-0.01, -0.001]	-0.005	0.003	-1.42	[-0.01, 0.002]
Burnout					0.10	0.02	6.56***	[0.07, 0.14]	0.19	0.02	11.81**	[0.16, 0.23]
Burnout×resilience					-0.003	0.002	-1.71	[-0.006, 0.00]	-0.003	0.002	-1.67	[-0.006, 0.00]
R^2^	0.50				0.39				0.52			
F	78.39***				36.37***				61.81***			

WFC: work-family conflict, *p < 0.05 (two-tailed), **p < 0.01 (two-tailed), ***p < 0.001 (two-tailed)

**Fig. 2:**
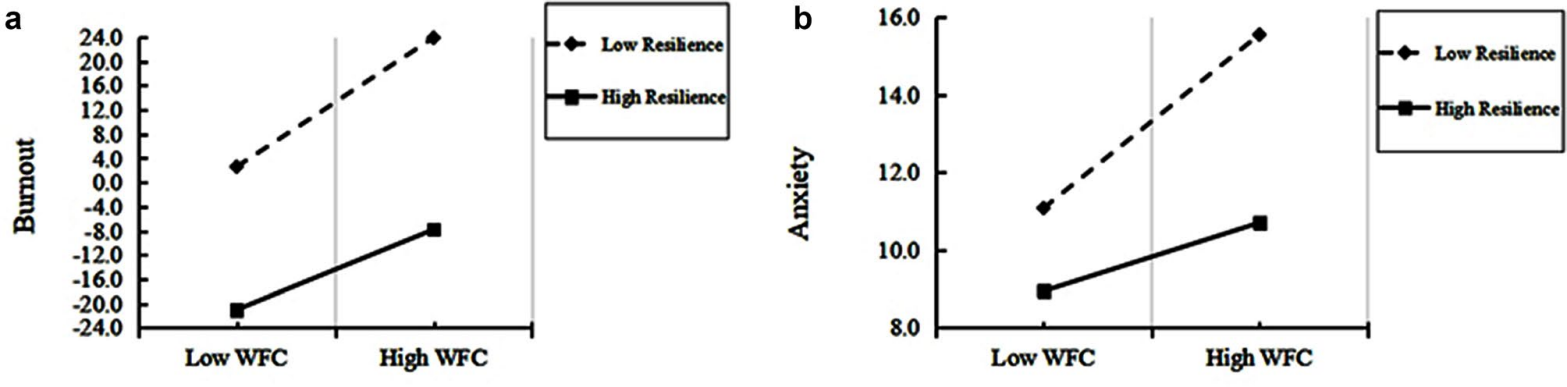
(**a**) Resilience as a moderator in the association between WFC and burnout. (**b**) Resilience as a moderator in the association between WFC and anxiety

Simple slope analyses were also conducted to evaluate the interaction effects. We first examined the predictive effect of WFC on burnout at one SD above and below the mean of resilience. The results showed that, when the level of resilience was low, WFC was positively predictive of burnout, with a simple slope b = 0.82 (p < 0.001); when the level of resilience was high, WFC was also positively predictive of burnout, but the predictive effect was lower, with a simple slope b = 0.51 (p < 0.001) (Fig. 2a). This suggested that with the increase of resilience, the predictive effect of WFC on burnout gradually reduces, i.e., higher resilience was associated with a lower mediating effect of burnout in the relationship between WFC and anxiety/depression. With regard to the predictive effect of WFC on anxiety at one SD above and below the mean of resilience, WFC was found to be significantly and positively predictive of anxiety when the level of resilience was low, with a simple slope b = 0.17 (p < 0.001); when the level of resilience was high, WFC was also found significantly and positively predictive of anxiety, but the predictive effect was relatively weaker, with a simple slope b = 0.07 (p < 0.05) (Fig. 2b). This indicated that higher resilience might weaken the predictive effect of WFC on anxiety.

## Discussion

Using moderation and mediation models, the present study explored the relationship between WFC and anxiety/depression as well as the underlying mechanism among correctional officers in China. The results showed that WFC was positively associated with anxiety and depression, and that burnout and resilience played a mediating and moderating role, respectively, in such a relationship. Resilience was found to moderate the direct effect of WFC on anxiety as well as the indirect effect of WFC on anxiety through mediation by burnout; it was also found to play a moderating role in the indirect effect of WFC on depression through mediation by burnout.

The present study demonstrates that greater WFC is linked to a high level of anxiety and depression, which supports our first hypothesis and is in line with prior studies [24, 41]. According to the role strain theory [42], individuals may not have enough time and energy to fulfill the responsibilities of multiple roles in their life, thus, taking on multiple roles simultaneously may lead to role conflicts, emotional tension and other negative outcomes [43]. In-service correctional officers often need to undertake both work and family roles, and frequent transition between different roles is likely to result in greater stress, which in turn induces anxiety and depression. From a practical point of view, correctional officers in China are exposed to a heavy workload due to the huge population base and limited workforce [2]. Correctional facilities must operate on a 24-hour basis daily throughout the year, therefore, correctional officers have to engage in shift work [12, 44]. As a result, correctional officers work long hours under tremendous pressure, leaving little time to fulfill family responsibilities, ultimately resulting in WFC. Furthermore, most in-service correctional officers are situated in the life stage characterized by familial responsibilities, including childcare and aged care. Consequently, these individuals may encounter challenges in maintaining their work-life balance, which results in increased psychological stress [45]. The increased stress caused by the accumulation of WFC may also lead to an altered level of serotonin, thereby resulting in psychological distress [46].

The present study demonstrates that WFC not only directly affects anxiety and depression but also indirectly affects the two symptoms via the mediation of burnout. In other words, individuals experiencing more WFC tend to experience greater burnout and ultimately develop psychological distress. This is consistent with previous findings that burnout played a mediating role in the association between work-related stress and anxiety/depression [47]. It has been suggested that WFC could reduce the sense of control and self-efficacy of individuals [48], making it difficult to maintain a positive self-image at work; this may lead to burnout and psychological distress. In line with the Job Demands-Resources (JD-R) model [19], individuals need a large amount of energy and resources to cope with accumulating stress caused by WFC, which can be exhausting and result in feelings of fatigue and helplessness (also known as emotional exhaustion) [19]. Individuals experiencing emotional exhaustion often feel overwhelmed, anxious, nervous, depressed or helpless [49]. Taken together, the above findings support the mediating role of burnout in the association between WFC and anxiety/depression.

The present study found that resilience played a moderating role in the first half of the effect of WFC on anxiety/depression through the mediation of burnout, i.e., a higher level of resilience might be able to buffer against the burnout caused by WFC, thereby relieving anxiety and depression. This result partially supported the third hypothesis of this study and indicated the protective nature of resilience [50]; this suggested that positive personal resources might help to reduce the impact of stressful events on the mental health of individuals [51]. Resilience has been referred to as the ability to utilize personal resources to restore the original psychological state in the presence of stress [52]. Previous studies have shown that individuals with a high level of resilience may have more personal resources and be prone to adopting more problem-focused coping strategies [53, 54], making them better at adapting to the environment and less likely to develop mental health problems. Therefore, the correctional officers with greater resilience might have actively and flexibly utilized personal resources to cope with WFC, thereby avoiding the development of burnout as well as anxiety and depression.

The present study found that resilience moderates the direct effect of WFC on anxiety, but not depression. These results are not completely consistent with the third hypothesis of this study and some previous findings [55]. The observed discrepancy can be attributed to the distinct nature, attributes and influencing factors of anxiety and depression as separate diseases. For instance, a study comparing generalized anxiety disorder (GAD) and major depressive disorder (MDD) reveals heightened emotional intensity and goal motivation in GAD and diminished positive affect in MDD [56]. Therefore, when faced with stressors, individuals experiencing anxiety may be more likely to utilize their personal resources to overcome difficulties, whereas individuals with depression may lack the energy or motivation to mobilize resources due to their low mood. The above-mentioned discrepancy might also be explained by resilience failing to buffer against certain subtypes of depression (such as endogenous depression), as prior findings have suggested resilience was not associated with anhedonia, a characteristic symptom of endogenous depression [57]. As the findings regarding the moderating effect of resilience on depression are still inconsistent, future studies are needed to examine the relationship between resilience and different subtypes of depression.

In the present study, we did not find a moderating effect of resilience on the association between burnout and anxiety/depression. This was inconsistent with the third hypothesis of our study and some previous findings that a higher level of resilience could buffer against anxiety and depression even in the presence of burnout [50]. Recently, resilience has been increasingly considered as a dynamic process that evolves over time and is influenced by personal characteristics, environment, family and social resources [58]. Thus, the environment and the controllability of adverse environments play an important role in the maintenance of resilience [52]. An individual’s attempts to alter an uncontrollable situation are unlikely to succeed and may result in a greater sense of frustration and desperation, thereby impairing their resilience [59]. Thus, some correctional officers in the present study might have developed burnout under tremendous pressure of work demand and family responsibility, and this uncontrollable situation might have resulted in their burnout, impaired resilience, and loss of function. Furthermore, it needs to be noted that the interaction between resilience and burnout may be influenced by multifaceted factors. Consequently, further studies are still needed to explore the role of resilience in the association between burnout and anxiety/depression.

Overall, the present study represents an initial endeavor to incorporate the mediating effect of burnout and the moderating effect of resilience to elucidate the relationship between WFC and psychological distress among Chinese correctional officers. In this study, we have identified a mediation effect of burnout on the association between WFC and anxiety/depression, as well as a moderating role of resilience in these relationships. These findings may provide evidence to support a connection between the essential factors of the J-DR model and the protective nature of resilience, thereby enhancing the theoretical framework regarding the mental well-being of correctional officers and improving our understanding of the mechanisms by which WFC impacts psychological distress.

## Implications

The findings of this study have significant practical implications for interventions. First, it highlights the negative effects of WFC on correctional officers, i.e., WFC may lead to burnout and psychological distress in this population, which needs more attention from policy-makers. For the correction officers, WFC can be better addressed by allocating more time for family and receiving family relationship management courses [6]. Moreover, resilience can buffer the impact of WFC on burnout and anxiety, thus, it is crucial to identify protective factors that improve resilience and implement psychological interventions to promote resilience [60], such as mindfulness based intervention [61] and resilience training [62], which emphasizes problem-solving skills and the reduction of catastrophic thinking.

## Limitations and future directions

First, the methodology of the present study was limited to self-reported questionnaires, which might have resulted in their providing of socially desirable responses, which might not accurately reflect their actual status. Future works may also involve mental health assessment of family members and colleagues of the correctional officers. Second, symptoms of anxiety and depression were assessed using self-report scales rather than standardized diagnostic instruments, which might have compromised the objectivity of the data. In future studies, structured psychiatric assessments should be used to accurately diagnose anxiety and depression of participants. Thirdly, the findings of this study are based on data collected from a sample of correctional officers in 29 provinces of China, who were recruited using a non-probability sampling method. Although our results may apply to a majority of regions in China, it is important to note that their applicability to other regions still requires additional validation. Thus, further studies with a larger sample encompassing a broader range of regions and using a random sampling method are warranted to improve the generalizability of the findings. Fourthly, data on specific categories of family matters impacting family life were not recorded, which precluded the analysis of their impact on WFC; thus, the impact of specific family matters may also be a focus of future research. Furthermore, gender differences in work and family roles cannot be ignored. Although our models included gender as a covariate, the power of the present study is still insufficient to examine gender differences. Therefore, future research can focus on the role of gender differences in WFC. Lastly, due to the cross-sectional nature, our study was not able to establish a causal relationship; thus, longitudinal studies are warranted to investigate the potential causality between WFC, burnout, resilience, and psychological distress.

## Conclusion

The present study demonstrates that WFC has both direct and indirect effects on the psychological distress of correctional officers, with the latter being mediated by burnout. It is also found that resilience may play a moderating role on part of the effect of WFC on anxiety/depression through the mediation of burnout and the direct effect of WFC on anxiety. In light of these findings, policymakers and practitioners involved in clinical intervention programs may need to prioritize the development of interventions to address WFC and improve resilience among correctional officers.

### Electronic supplementary material

Below is the link to the electronic supplementary material.

Supplementary Material: A figure of the geographic distribution of the participants

## Data Availability

The datasets used and/or analyzed during the current study are available from the corresponding author on reasonable request.

## Abbreviations

WFC: work-family conflict
WFCS: Work-Family Conflict Scale
DASS: Depression Anxiety Stress Scale
MBI-GS: Maslach Burnout Inventory-General Survey
CD-RISC: Connor-Davidson Resilience Scale
JD-R model: Job Demands-Resources model
SCID-5: Structured Clinical Interview for DSM-5
GAD: Generalized anxiety disorder
MDD: Major depressive disorder
