# Supplementary figures and images for: Relationship between work-family conflict and anxiety/depression among Chinese correctional officers: a moderated mediation model of burnout and resilience
Source: BMC Public Health. 2024 Jan 2;24:17. doi: 10.1186/s12889-023-17514-6 (PMC10759684; doi:10.1186/s12889-023-17514-6)

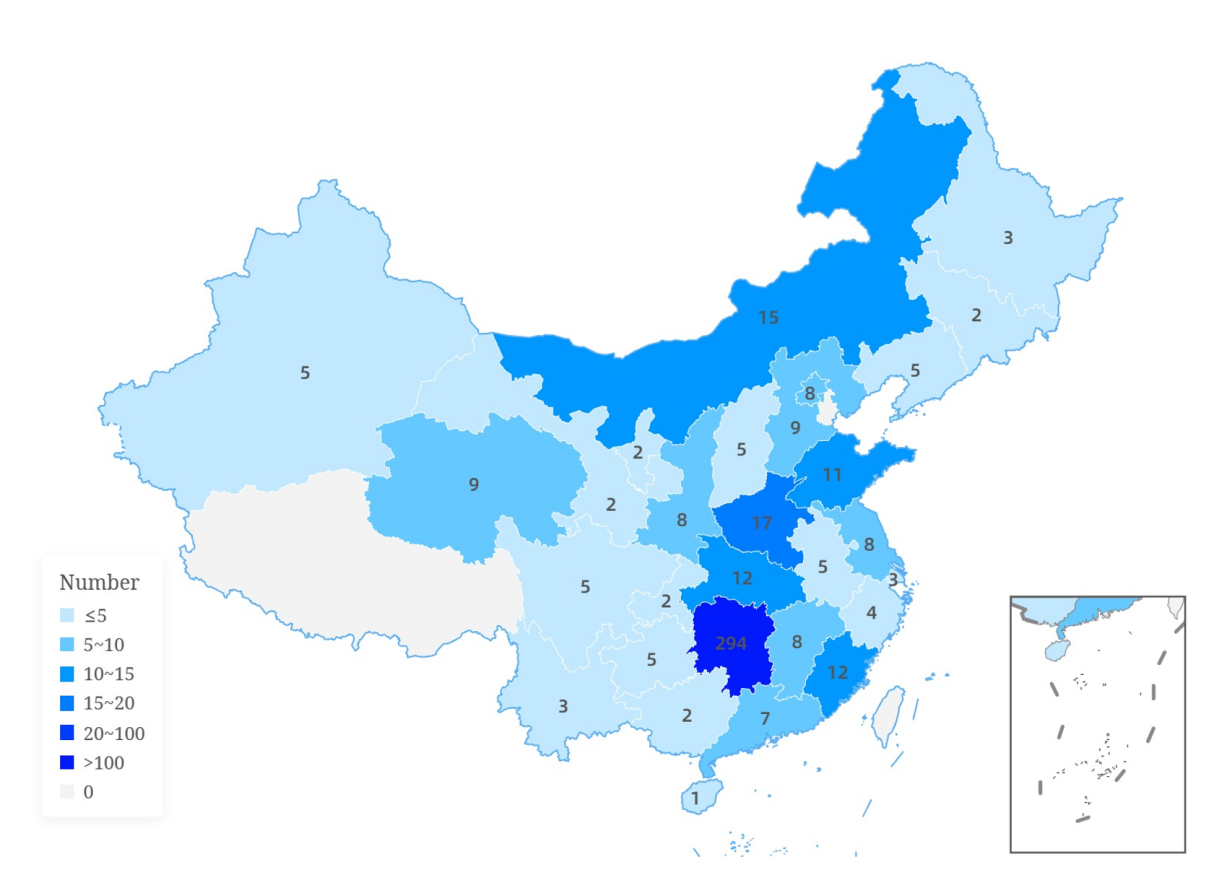


**Figure S1 The geographic distribution of the participants**

Supplement: Supplementary file 1 — Supplementary Material: A figure of the geographic distribution of the participants [file 12889_2023_17514_MOESM1_ESM.docx]
